# Optimization of Dietary Zinc Requirement for Broiler Breeder Hens of Chinese Yellow-Feathered Chicken

**DOI:** 10.3390/ani9070472

**Published:** 2019-07-23

**Authors:** L. Li, K. F. M. Abouelezz, Z. Gou, X. Lin, Y. Wang, Q. Fan, Z. Cheng, F. Ding, S. Jiang, Z. Jiang

**Affiliations:** 1 College of Animal Science, South China Agricultural University, Guangzhou 510642, China; 2 Institute of Animal Science, Guangdong Academy of Agricultural Sciences, Guangzhou 510640, China; 3 State Key Laboratory of Livestock and Poultry Breeding, Guangzhou 510640, China; 4 Key Laboratory of Animal Nutrition and Feed Science in South China, Guangzhou 510640, China; 5 Ministry of Agriculture, Guangdong Public Laboratory of Animal Breeding and Nutrition, Guangzhou 510640, China; 6 Guangdong Key Laboratory of Animal Breeding and Nutrition, Guangzhou 510640, China; 7 Department of Poultry Production, Faculty of Agriculture, Assiut University, Assiut 71526, Egypt

**Keywords:** Zn requirement, productive and reproductive performance, egg quality, tibial quality, antioxidant enzymes, Chinese yellow-feathered broiler breeder hens

## Abstract

**Simple Summary:**

China is the second-largest global producer of chicken meat, almost half of which is from the Chinese yellow-feathered breed; a systematic program has been initiated to improve its feeding standards. This study evaluated the optimal requirement of dietary zinc for maximal egg production, egg quality, tibial quality, and antioxidant indices of laying broiler breeders. The results revealed several beneficial effects of supplementary zinc on egg production, feed conversion ratio, yolk zinc content, tibial quality and the antioxidant indices in the serum, liver and ovary. The optimal zinc requirement was estimated based on a regression model.

**Abstract:**

This study aimed to establish the optimal dietary zinc requirement of Chinese yellow-feathered Lingnan broiler breeders. A total of 576 breeder hens aged 58 weeks were randomly assigned to six treatments, each with 6 replicates of 16 birds (*n* = 96/treatment). The hens were fed either a basal diet (22.81 mg/kg Zn) or the same basal diet supplemented with additional 24, 48, 72, 96, and 120 mg Zn/kg up to 65 weeks of age. Compared to the results of birds fed the basal diet (22.81 mg Zn/kg), the dietary supplementation with additional Zn (mg/kg) showed higher egg laying rate (at 48–120 mg), EM (at 96 mg/kg), yolk Zn content (at 24–120 mg/kg), fertility (at 48–120 mg/kg), hatchability (at 48–96 mg/kg), tibial breaking strength (at 24–48 mg/kg), tibial ash content (at 48 mg/kg), serum CuZnSOD activity (at 72 mg/kg) and T-AOC (at 48 mg/kg), and ovarian CuZnSOD and GSH-Px activities (at 96–120 mg/kg), and lower FCR (at 96 mg/kg). The regression model showed that the optimal supplemental Zn for maximal egg laying rate, yolk Zn content, fertility, and hatchability of Chinese yellow-feathered broiler breeders aged 58 to 65 weeks were 71.09, 92.34, 94.44 and 98.65 mg/kg diet, respectively.

## 1. Introduction

Zn is an important trace mineral, which is involved in various biological activities in animals’ bodies [1,2]. It is considered to be a coenzyme of more than 240 enzymes in birds’ bodies, and plays a major role in activating some antioxidant enzymes which sustain the defense system of avian body against reactive oxygen species [1,2,3]. The results of previous studies have indicated that Zn supplementation is important for enhancing the antioxidant capacity and improving the productive and reproductive performance [4,5,6,7], egg quality [8,9,10], and tibial quality [11,12,13] in laying hens and laying breeders. Reproductive performance, egg quality, tibial quality, antioxidant status, and tissue deposition are common variables employed in estimating the optimal dietary nutrient requirements for laying hens and breeders, following nonlinear regression models [6,14].

Chinese yellow-feathered chickens contribute around 50% of the chicken meat produced in China, the second-largest global producer of chicken meat, with annual production exceeding four billion birds of this breed, which is comparable to the population of white-feathered broiler hybrids in China [15]. There is an increasing commercial importance of these indigenous birds due the distinct flavor of their meat; however, comprehensive work is still needed to improve their feeding standards and those of their breeders. Zn requirement of Chinese yellow-feathered laying hens was 72 mg/kg diet, as indicated by Feeding Standard of Chicken [16], but these data were formulated more than 15 years ago and likely do not meet the actual needs of the modern strain of such birds, which has been subjected to a program of genetic improvement to meet the aspirations of poultry production industry. Additionally, the previous research on Zn requirements of laying hens was conducted in white-egg laying hens, which differ than those of the Chinese yellow-feather chicken breeders [6,14]. The aim of this study, therefore, was to estimate the dietary Zn requirements for optimal productive and reproductive performance, egg quality, tibial quality, yolk Zn deposition, and antioxidant status of Chinese yellow broiler breeder hens.

## 2. Materials and Methods

### 2.1. Birds, Diet and Management

All experimental methods in this work conformed to the guidelines of the Animal Care and Use Committee, Guangdong Academy of Agricultural Sciences (GAASIAS-2016-2017). A total of 576 fifty-eight week-old Chinese yellow-feathered broiler breeder hens (Lingnan, an improved local breed) were obtained from Guangdong Wiz Agricultural Science & Technology Co. Ltd. (Guangzhou, China) and used in this study. Birds with a similar body weight (BW) (3142.50 g) and egg laying rate (52%) (ELR) averages were assigned randomly to six treatment groups, each treatment (*n* = 96 birds) consisted of six replicates with 16 birds each, which were housed in 8 doubled cages (40 cm × 45 cm × 45 cm). The ZnSO_4_·H_2_O was added to a basal diet to obtain six levels of Zn (0, 24, 48, 72, 96 or 120 mg/kg, calculated), which were then pelleted and provided daily (118 g/bird) during the 8 weeks (wk) of experimental period from 58 to 65 wk of age. The basal diet (Table 1) was formulated to meet or exceed the nutritional requirements of broiler breeder hens (Ministry of Agriculture, China, [16]) except for Zn (22.81 mg/kg). During the experimental period, the house temperature ranged from 22 to 27 °C, water was provided ad libitum, and the lighting regimen was 16 h light per day, from 06:00 to 22:00.

At the end of the experimental period (at 65 week of age), at 9:00 AM after overnight fasting for 14 h, two birds from each replicate (*n* = 12/treatment) were randomly chosen, and blood samples were collected from the left-wing vein using 5 mL vacutainer non-heparinized tubes. Within 30 min of collection, the serum samples were separated by centrifugation (1200× *g* for 15 min) and stored in 0.5 mL Eppendorf tubes at −20 °C until analysis. The birds were then weighed, euthanized by cervical dislocation, and exsanguinated and the tibia, liver, ovaries, oviducts and large follicles were collected. The ovaries and oviducts were weighed and the oviduct length, and number and weight of total large follicles (>8 mm) were determined according to Johnson [17].

### 2.2. Productive Performance

On a replicate basis, the final BW of hens at 65 week of age, feed intake, average daily gain, ELR (%), average daily feed intake, average egg weight, daily egg mass (EM), feed conversion ratio (g feed: g egg, FCR), and sellable egg percentage (%) were determined from 58 to 65 week of age.

### 2.3. Egg Incubation Indices

During wk 64 of age, all birds were inseminated artificially two times, with a three days interval, with 100 µL of fresh semen (diluted 1:1 volume/volume, with 0.9% saline solution) collected from males belonging to the same breed. During the following wk (65th wk), fifty settable eggs (of 50–70 g weight) were collected from each replicate (*n* = 300 eggs/treatment), weighed and incubated under standard incubation conditions (at 36.5 °C to 38.4 °C, and 55% to 65% relative humidity). At hatch, all unhatched eggs were cracked to identify the unfertile eggs and to calculate the fertility rate (%) [(Number of fertile eggs/number of total incubated eggs) × 100]. The hatchability rate (%) [(Number of hatched chicks/number of incubated eggs) × 100], the sellable chick percentage (%) [(Number of healthy hatched chicks/number of fertilized eggs) × 100], and the average chick weight (g) at birth were calculated.

### 2.4. Tibial Quality Measurements

The length and fresh weight of collected tibias, free of fascia tissues, were measured. The left tibias were used in measuring the breaking strength, which was performed using the tenderness tester (INSTRON 4411, Instron Corporation, Norwood, MA, USA) as described by Chen et al. [18]. Right tibias were boiled in water for 6 min and de-fatted by soaking in diethyl-ether for 96 h, which were then oven dried to a constant weight and ashed using a muffle furnace (550–600 °C for 24 h) to determine the ash content, expressed on the basis of dry-defatted weight. The obtained ash of the right tibias was used in analyzing the content of Zn by flame atomic absorption spectrometry (SpectrAA50/55, Warian Corporation, Palo Alto, CA, USA).

### 2.5. Egg Quality Indices

During the week 64 of age, a total of 504 eggs (2 eggs per replicate/day), were collected, labelled, weighed individually, and used in measuring the egg quality indices. Egg shell strength was determined with an Egg Force Reader (EFR-01, Orka, Ramat HaSharon, Israel). Egg shape index [(width/length) × 100], shell thickness (average thickness at the blunt end, sharp end and middle points of the egg), yolk color, and Haugh unit were measured using an egg multi tester EMT-5200 (Robotmation Co. Ltd., Tokyo, Japan). Yolk, shell (free of membranes), and albumin fractions of the total egg weight (%) were determined.

### 2.6. Biochemical Determinations of Liver, Ovary and Serum

Homogenates of liver and ovary tissues, prepared as described by Zhang et al. [5], were clarified by centrifugation at 3000× *g* for 10 min at 4 °C and the supernatants were used in enzyme assays. In serum, liver and ovary samples, content of malondialdehyde (MDA), total antioxidant capacity (T-AOC), and glutathione (GSH) were determined. The activity of glutathione peroxidase (GSH-PX), copper-Zn superoxide dismutase (CuZnSOD), and alkaline phosphatase (AKP) were measured using kits purchased from Nanjing Jiancheng Bioengineering Institute, Nanjing, China, and using an automated spectrophotometric analyzer (Cobas FARA II, Roche, Palo Alto, CA, USA) according to the manufacturer’s directions.

### 2.7. Zn Content in Diet, Serum, Tibia and Yolk

Concentrations of Zn in diets, serum, tibia and yolk were measured by flame atomic absorption spectrometry (SpectrAA50/55, Warian Company of America) as described by Liao et al. [19].

### 2.8. Statistical Analysis

The effects of dietary treatment were assessed by one-way GLM ANOVA procedures of SAS (version 9.3, SAS Inst., Cary, NC, USA, 2014), and replicates were considered to be as the experimental unit for each variable (*n* = 6). When not presenting residues with normal distribution, data were transformed with the arcsine square root percentage (z = asin (sqrt (y + 0.5))) [20]. All data were expressed as means and SEM, derived from the ANOVA error mean square. Estimates of Zn requirements were obtained using quadratic polynomial (QP) or exponential asymptotic (EA) regression analysis [21,22]. Where appropriate, quadratic polynomial (Y = c + bX + aX2; where “Y” is the dependent variable as a function of dietary level of Zn, “c” is the intercept, “b” is the linear coefficient, and “a” is the quadratic coefficient.) and exponential asymptotic [Y = a + b × (1 − EXP (−c × (X − d))); where “Y” is the dependent variable as a function of dietary level of Zn, “a” is the relative response to the diet containing the lowest Zn, “b” is the difference between the minimum and the maximum response dietary Zn, “c” is the curve slope coefficient, and “d” is the Zn level of the deficient diet] models were fitted to the responses of the dependent variables to Zn level. The level of dietary Zn at which the response reached 95% at maximum was estimated as the requirement.

## 3. Results

### 3.1. Productive Performance

The effects of dietary Zn levels on Lingnan breeder hens’ BW and egg production variables are shown in Table 2. The ELR at 48 to 120 mg Zn/kg diets were higher (*p* < 0.01) than those of 24 mg/kg and controls. The birds fed 96 mg of supplemental Zn/kg diet showed a higher daily EM and lower FCR compared to the controls (*p* < 0.05). There were no significant differences in the final BW, average daily gain, sellable egg rate, and average egg weight (*p* > 0.05) due to the dietary Zn concentration. The regression model indicated that the optimal Zn level for maximal ELR was 71.09 mg Zn/kg diet.

### 3.2. Egg Quality Indices

The results shown in Table 3 indicate that the egg quality indices, in terms of egg weight, egg shape index, eggshell strength, eggshell thickness, and Haugh unit were not affected (*p* > 0.05) by the dietary Zn level. The content of Zn in egg yolks increased (*p* < 0.05) in all treatments received additional dietary Zn than in controls and, according to the regression model, the maximal yolk Zn content was obtained with 92.34 mg of supplemental Zn/kg diet.

### 3.3. Egg Incubation Indices

As shown in Table 4, the fertility, hatchability and salable chick rate values increased significantly (*p* < 0.05) with 48, 72 and 96 mg of supplemental Zn/kg (*p* < 0.05) compared to controls. The chick weight at birth was not affected by the supplemental Zn (*p* > 0.05). According to the regression model, the optimal levels of supplemental Zn for fertility and hatchability were 94.44 and 98.65 mg/kg diet.

### 3.4. Tibia Quality

There was a significant effect of dietary Zn level on the tibial breaking strength and tibial ash content of breeder hens (Table 5). Hens fed diets with an additional 24 and 48 mg/kg Zn both had higher (*p* < 0.05) tibia breaking strength compared to that of the controls and 72 mg Zn/kg treatment. Ash content of the tibia was higher (*p* < 0.05) in breeders received 48 mg additional Zn/kg diet than in those fed the basal diet and 72 mg additional Zn/kg.

### 3.5. Ovarian and Oviductal Variables

The results shown in Table 6 reveal that the ovarian weight, oviduct weight, oviduct length, and the numbers and weights of dominant follicles were not affected by the additional Zn levels (*p >* 0.05).

### 3.6. Biochemical Variables in Serum, Liver and Ovary

As shown in Table 7, the birds fed 72 mg supplemental Zn/kg exhibited significantly increased CuZnSOD activity in serum (*p* < 0.05) compared with those in the other treatments. The dietary addition of Zn at levels 48 to 120 mg/kg diet showed lower serum MDA content than those of the lower levels. The T-AOC and Zn content of serum in hens supplemented with 48 and 96 mg Zn/kg diet were higher (*p* < 0.05) than the corresponding values in the other treatments, respectively. The activities of CuZnSOD in the liver of hens fed a diet with 48, 72, 96, and 120 mg of supplemental Zn/kg was higher than those in the controls (*p* < 0.01). The supplemental Zn levels did not show significant effects on AKP activity, GSH content and GSH-Px activity in serum, or MDA content in the liver tissue (*p* > 0.05). The results, in Table 8, showed that the biochemical indices in the ovary of Chinese breeder chickens as affected by dietary Zn levels. The birds fed 96 and 120 mg supplemental Zn/ kg diet showed higher (*p* < 0.01) CuZnSOD activity and GSH-Px activity in the ovary tissues than those in controls. The MDA, GSH and T-AOC of the ovary were not affected by the additional dietary Zn (*p* > 0.05).

## 4. Discussion

The findings of the present study confirm that the tested dietary Zn levels affected the egg production, reproductive performance, biochemical levels, ovary and liver, and tibial quality of Chinese yellow chicken breeders. Supporting results were reported in previous studies. Zhang et al. [5] found that the dietary addition of 80 mg supplemental Zn/kg of a basal diet containing 24 mg Zn/kg (104 mg/kg as a final concentration) improved the FCR, ELR, fertility and hatchability of Lingnan Yellow broiler breeders from 38–57 week of age. Naibi et al. [23] showed that supplementing the Yuehuang broiler breeders with 70.09 mg Zn/kg of corn-soybean diets from 14 to 34 week of age increased their ELR, fertility and hatchability than those in the controls. Mayer et al. [14] found that the Cobb 500 broiler breeder hens fed diets containing 50.3 to 170.6 mg Zn/kg between 37 and 40 week of age showed a higher ELR than in controls. Other studies showed partial consistency with our results. The results of Durmuşg et al. [24] indicated that the brown laying hens fed 180 mg Zn/kg had the highest hatchability, whereas those fed a diet supplemented with 120 mg Zn/kg had the lowest FCR than the controls and other treatments. Sharideh et al. [4] found that the Cobb 500 broiler breeder hens fed diets containing 90 and 120 mg Zn/kg showed a higher fertility rate than those of birds fed 30 and 60 mg Zn/kg between 62 and 72 week of age. In White Leghorn hens, Stahl et al. [25] concluded that the diet with 28 mg Zn/kg was sufficient to prevent decreased egg production, fertility, hatchability and growth of progeny. 

The results obtained here, compared to the controls, revealed higher ELR and fertility with the levels from 48 to 120 mg of supplemental Zn/kg (70.81 to 142.81 mg Zn/kg, as final concentrations), higher daily EM and lower FCR with 96 mg of supplemental Zn/kg (118.81 mg/kg, as a final concentration), and a higher hatchability with the levels from 48 to 96 mg of supplemental Zn/kg (70.81 to 118.81 mg Zn/kg, as final concentrations). The results obtained in the present study as well as the aforementioned findings of previous studies therefore show that the response of broiler breeders to dietary Zn is dependent on the supplemental levels, the measured variable, the genetic differences, the physiological status of the bird, and age. 

Egg quality has been reported to be an important indicator in the evaluation of the optimal dietary Zn level for laying hens [8,25]. The results here showed that there was no effect of dietary Zn concentration on all egg quality measurements, but the hens fed 48 mg of supplemental Zn/kg diet (70.81 mg Zn/kg, as final concentration), or higher, had a higher yolk Zn content. Our results were consistent with those of Mayer et al. [14], and agreed partially with those of Bahakaim et al. [9], where the addition of 50, 100 and 150 mg of Zn/kg did not affect the egg shape index, shell thickness, shell, yolk and albumen fractions of the egg, but the Haugh unit decreased significantly at 150 mg Zn/kg compared to the controls. Nutritional status of laying breeders ensures adequate nutrient transferee into the egg, which is required for a normal development of embryos [26]. Among the egg components, the yolk is considered to be the major mineral source for the embryo, which contains most of the mineral content of the egg, including the P, Zn, Cu, Mn and Fe [27]. Badawy et al. [28] reported a positive relationship between Zn content in the egg and hatchability values. This could explain the consistency between the obtained increase in yolk Zn content and the corresponding increase in fertility and hatchability in the present study. 

Bone status is a common variable used in estimating mineral adequacy in poultry diets [29]. Zn is essential for bone strength; it enters in the formation of bone tissue in the form of alkaline phosphatase, collagenase and aminocly tRNA synthetase [30,31,32,33,34]. The insufficient dietary Zn caused a deterioration of bone formation, which reduced bone density due to its important role in protein synthesis [33,35,36]. The results of the present study showed that the dietary addition of 24 and 48 mg of supplemental Zn (46.81 and 70.81 mg/kg as final concentration) increased the tibial breaking strength (*p* < 0.01) than those in the control (22.81 mg Zn/kg). The result obtained here is consistent with the previous finding of Olgun et al. [10], who reported that using 75 mg Zn/kg diet significantly increased the shear force of the tibia when compared to values obtained with 50 or 100 mg Zn/kg. Stofaníková et al. [12] found that birds fed 100 mg Zn/kg diet had higher tibia strength compared to chickens fed 50 mg/kg group. The tibial ash content reflects the rate of the mineral density of bones [37], which affects bone strength [38]. Our data showed that the tibial ash content of birds received 48 mg of supplemental Zn/kg diet (70.81 mg Zn/kg, as a final concentration) was higher than those of the control (fed 22.81 mg/kg). This is in agreement with the results reported previously by Ao et al. [11] and Sahraei et al. [13]. The results here, therefore, suggest that the 70.81 mg Zn/kg diet (as a final concentration) is an adequate level for laying breeders, which led to a higher breaking strength and ash content of the tibia.

The enzyme CuZnSOD exerts an important function in maintaining the redox balance of the bird’s immune system by eliminating reactive oxygen species [39]. Zn is an important functional constituent of that enzyme (CuZnSOD), which accounts for around 90% of its structure [3,39]. The total antioxidant capacity (T-AOC) contributes mainly to the dynamic balance of active oxygen, which works as an integrative factor reflecting the status of all antioxidants in serum and body fluids [40]. MDA content is used as an indicator of lipid peroxidation and oxidative damage caused by reactive oxygen species [41]. The present study showed that the dietary Zn supplementation (mg/kg diet) increased the activity of serum CuZnSOD (with 72 mg/kg) and T-AOC (at 48 mg/kg) content, and the dietary levels from 48 to 120 mg/kg reduced the serum content of MDA and increased the activity of CuZnSOD in the liver as compared to the controls. Supporting results were reported by Zhang et al. [5], who found that using 104 mg Zn/kg increased the antioxidant status by stimulating the activity of CuZnSOD and T-AOC in the serum, and suppressed the generation of ROS, and therefore decreased MDA content in hen’s serum and liver. In a similar manner, Zhao et al. [40] found that the addition of Zn to the feed increased T-AOC capacity and CuZnSOD activity in serum. 

In the poultry industry, the age-related decline in reproductive performance of breeder stocks is a common phenomenon [42,43,44]. Jiang et al. [45] and Liu et al. [46] reported that the low antioxidant status in the ovary is associated with age-related decline in hen’s reproduction. Liu et al. [46] found that enhancing the antioxidant enzyme activities can effectively prevent the ovarian aging process in hens. This indicates that the results obtained here regarding the enhanced ovarian CuZnSOD and GSH-Px activities due to Zn supplementation has a significant importance, where our experiment was carried out with breeders at the late phase of the laying period. This could imply that the dietary Zn supplementation could contribute in alleviating the decline in reproductive performance of breeders at late ages through increasing the antioxidant activities and, therefore, persist a longer productive season with higher performance; this is confirmed by the improved ELR and EM in the present study. 

Evaluating the response of more than one variable to a dietary nutrient makes it difficult to determine a unique requirement value [14]. Pesti et al. [22] reported that the nutritional requirements derived from dose-response experiments are considered physiologically less accurate than regression models, since it hypothesize symmetrical fixed responses to deficiency and excess, but the non-linear models depict the biological responses better than models that force responses to conform to straight lines. Additionally, Mayer et al. [14] reported that using QP, broken line quadratic (BLQ), and EA models was more appropriate for Zn requirements in Cobb 500 broiler breeder hens [14]. In the present study, we therefore used QP Models and EA models in determining the optimal levels of Zn for the most important indices. The recommended Zn requirement according to the Feeding Standard of Chicken [16] for Chinese yellow-feathered laying hens is 72 mg Zn/kg of feed. According to the QP models and EA models, the estimated optimal Zn requirement for broiler breeders were 71.09, 92.34, 94.44 and 98.65 mg/kg, for ELR, yolk Zn content, fertility and hatchability, respectively. The value estimated here for ELR was close to the recommended value of Feeding Standard of Chicken [16] for laying hens, and those estimated for yolk Zn content, fertility and hatchability were higher than the corresponding values in the Feeding Standard of Chickens [16]. 

## 5. Conclusions

Compared to the results of the birds fed the basal diet containing 22.81 mg Zn/ kg, the dietary supplementation with additional Zn (mg/kg) showed higher ELR (at 48 to 120 mg/kg), EM (at 96 mg/kg), yolk Zn content (at 24 to 120 mg/kg), fertility (at 48–120 mg/kg), hatchability (at 48–120 mg/kg), salable chick rate (at 48–96 mg/kg), tibial breaking strength (at 24 and 48 mg/kg), tibial ash content (at 48 mg/kg), serum CuZnSOD activity (at 72 mg/kg) and T-AOC (at 48 mg/kg), CuZnSOD activity in the liver (at 48–120 mg/kg), and ovarian CuZnSOD and GSH-Px activities (at 96–120 mg/kg), and lower FCR (at 96 mg/kg) and serum MDA content (at 48–120 mg/kg). The regression model indicated that the estimated requirements of supplemental Zn for maximal ELR, yolk Zn content, fertility, and hatchability of Chinese yellow-feathered broiler breeder hens aged 58 to 65 wk were 71.09, 92.34, 94.44 and 98.65 mg/kg diet, respectively.

## Figures and Tables

**Table 1:** Composition of the basal diet (air-dry basis, %).

Ingredient	Amount	Nutritional Level ^2^	Value
Corn	58.96	ME (kcal/kg)	2770
Soybean meal	24.76	CP (%)	16.36
Soybean oil	3.20	Methionine (%)	0.40
DL-Methionine	0.16	Lysine (%)	0.83
Limestone powder	7.2	Non-phytate phosphorus (%)	0.41
Dicalcium phosphate	1.83	Calcium (%)	3.03
Salt (Na Cl)	0.25	Zinc ^3^ (mg/kg)	22.81
Corn core poweder	2.64		
Premix ^1^	1.00		
Total	100		

^1^ Provided the following per kg of diet: vitamin A, 15,000 IU; vitamin D3, 3600 IU; vitamin E, 53 IU; vitamin K3, 6 mg; thiamin, 3 mg; riboflavin, 9 mg; pyridoxine, 6 mg; cyanocobalamin, 0.03 mg; pantothenic acid, 18 mg; niacin, 60 mg; folic acid, 1.5 mg; biotin, 0.18 mg; choline, 600 mg; Fe, 72 mg; Cu, 7.2 mg; Mn, 90 mg; Zn, 0 mg; I, 0.9 mg; Se, 0.48 mg. ^2^ Values were calculated based on the data provided by Feeding Standard of Chicken (Ministry of Agriculture, China, 2004). ^3^ Calculated Zn content based on Zn analyses in Corn, soybean protein concentrate, calcium carbonate, di-Ca phosphate, Corn core powder. Zinc was analyzed by atomic absorption spectrophotometry. The analyzed value of the dietary zinc was 22.81 mg/kg.

**Table 2:** Effects of dietary zinc supplementation on productive performance of Chinese yellow-feathered broilers breeders aged 58 to 65 weeks.

Variables	Dietary Zinc Supplementation (mg/kg) ^1^						SEM ^2^	*p*-Value
	0	24	48	72	96	120		
Initial body weight (g)	3125.83	3145.83	3147.5	3152.5	3129.16	3154.16	5.07	0.473
Final body weight (g)	3349.16	3386.66	3368.17	3330.5	3243.83	3370	18.7	0.281
Average daily gain (g)	2.79	3.11	3.04	2.88	2.45	2.66	0.2	0.172
Average daily feed intake (g)	118	118	118	118	118	118	0.0	1.000
Egg laying rate (%)	44.15 ^b^	46.11 ^a,b^	49.23 ^a^	51.99 ^a^	52.53 ^a^	51.52 ^a^	0.82	0.006
Average egg weight (g)	63.32	63.60	64.15	61.97	63.21	63.05	0.31	0.501
Daily egg mass (g egg/bird)	31.04 ^b,c,d^	29.31 ^d^	30.25 ^c,d^	31.81 ^a,b,c^	33.17 ^a^	32.18 ^a,b^	0.27	<0.001
Feed conversion ratio (g feed: g egg)	3.98 ^a,b^	4.17 ^a^	4.02 ^a,b^	3.74 ^b^	3.61 ^c^	3.77 ^a,b,c^	0.038	<0.001
Sellable egg percentage (%)	95.26	97.09	96.18	94.10	95.62	95.18	0.43	0.441
Exponential asymptotic regression model of Laying rate ^3^	Equations Y = 38.24 + 15.61 × (1 − EXP (−0.0176× (X − 22.81)))				*R* ^2^	*p*	ER ^4^, mg/kg	
					0.80	0.014	71.09	

In the same row, means not sharing a similar superscript (^a,b,c,d^) differ significantly (*p* < 0.05), the number of replicates was used as the experimental unit (*n* = 6). ^1^ The “0” treatment contained 22.81 mg Zn/kg, and the other zinc levels are additional to that level. ^2^ Standard error of the mean from ANOVA (*n* = 6), ^3^ Y is laying rate, and X is total dietary content of zinc. ^4^ ER: Estimated from regression analysis.

**Table 3:** Effects of dietary zinc supplementation on egg quality of yellow-feathered broiler breeders aged 58 to 65 weeks.

Variable	Dietary Zinc Supplementation (mg/kg) ^1^						SEM ^2^	*p*-Value
	0	24	48	72	96	120		
Egg shape index	1.35	1.33	1.32	1.30	1.33	1.33	0.005	0.268
Eggshell strength (N)	3.58	3.66	3.78	3.67	3.81	3.72	0.075	0.953
Egg thickness (mm)	0.31	0.32	0.33	0.31	0.32	0.31	0.026	0.282
Haugh unit	69.03	69.85	71.93	67.64	64.89	68.41	1.29	0.234
Yolk (%)	32.61	32.57	31.84	31.67	31.39	31.18	0.357	0.252
Shell (%)	8.63	9.06	9.32	9.06	8.76	8.98	0.09	0.909
Yolk zinc content (mg/kg)	24.00 ^b^	29.83 ^a^	30.33 ^a^	30.17 ^a^	30.00 ^a^	28.17 ^a^	0.357	0.043
Quadratic polynomial regression model of yolk zinc content ^3^	Equations				*R* ^2^	*p*	ER ^4^ mg/kg	
	Y = 20.31 + 0.23X − 0.0013X^2^				0.47	<0.0001	92.34	

In the same row, means not sharing a similar superscript (^a,b^) differ significantly (*p* < 0.05), the number of replicates was used as the experimental unit (n = 6). ^1^ The “0” treatment contained 22.81 mg Zn/kg, and the other zinc levels are additional to that level. ^2^ Standard error of the mean from ANOVA (*n* = 6). ^3^ Where Y is yolk zinc, and X is total dietary content of zinc; ^4^ ER, Estimated from regression analysis.

**Table 4:** Effects of dietary zinc supplementation on reproductive performance of yellow-feathered broiler breeders aged 65 weeks.

Variable	Dietary Zinc Supplementation (mg/kg) ^1^						SEM ^2^	*p*-Value
	0	24	48	72	96	120		
Fertility ^3^ (%)	82.75 ^b^	89.32 ^a,b^	96.99 ^a^	95.51 ^a^	96.26 ^a^	91.24 ^a^	1.27	0.023
Hatchability ^4^ (%)	72.69 ^b^	81.30 ^a,b^	94.21 ^a^	90.54 ^a^	92.57 ^a^	85.28 ^a,b^	2.21	0.045
Salable chick rate (%) ^5^	68.99 ^b^	78.41 ^a,b^	93.63 ^a^	88.56 ^a^	91.22 ^a^	85.28 ^a,b^	2.91	0.031
Chick weight at birth (g)	41.78	42.87	42.22	42.20	43.23	42.04	0.085	0.451
Quadratic polynomial regression model of fertility ^6^	Equations			R^2^	*p* value	ER ^7^ (mg/kg)		
	Y = 72.17 + 0.51X − 0.0027X^2^			0.37	0.0005	94.44		
Quadratic polynomial regression model of hatchability ^6^	Y = 57.42 + 0.73X − 0.0037X^2^			0.83	0.0066	98.65		

In the same row, means not sharing a similar superscript (^a,b^) differ significantly (*p* < 0.05), the number of replicates was used as the experimental unit (n = 6). ^1^ The “0” treatment contained 22.81 mg zinc/kg, and the other zinc levels are additional to that level. ^2^ Standard error of the mean from ANOVA (n = 6). ^3^ Fertility (%) = (number of fertile eggs/number of total egg set) × 100. ^4^ Hatchability (%) = (number of chicks hatched/number of eggs set) × 100. ^5^ Salable chick (%) = (number of healthy hatched chicks/number of fertilized eggs) × 100. ^6^ Y is fertilization rate or hatchability, and X is total dietary content of zinc. ^7^ ER: estimate from regression analysis.

**Table 5:** Effects of dietary zinc supplementation on tibia indices of yellow-feathered broiler breeders aged 65 weeks.

Variable	Dietary Zinc Supplementation (mg/kg) ^1^						SEM ^2^	*p*-Value
	0	24	48	72	96	118		
Breaking strength (kgf)	198.98 ^b^	267.90 ^a^	281.39 ^a^	232.66 ^b,c^	252.66 ^a,b,c^	256.08 ^a,b,c^	6.51	<0.001
Fresh weight (g)	18.96	19.74	21.66	19.53	19.86	20.01	0.30	0.179
Longitude (mm)	9.45	9.55	9.57	9.56	9.20	9.32	0.48	0.352
Dry weight (g)	9.71	11.22	12.45	10.69	11.04	11.73	0.39	0.146
Ash content ^3^ (%)	65.21 ^b^	66.64 ^a,b^	70.29 ^a^	66.08 ^b^	67.84 ^a,b^	68.37 ^a,b^	0.67	0.045
Zinc content (mg/kg ash)	200.00	225.00	243.33	222.00	218.33	238.33	4.75	0.094

In the same row, means not sharing a similar superscript (^a,b,c^) differ significantly (*p* < 0.05), the number of replicates was used as the experimental unit (*n* = 6). ^1^ The “0” treatment contained 22.81 mg zinc/kg, and the other zinc levels are additional to that level. ^2^ Standard error of the mean from ANOVA (*n* = 6). ^3^ On dry matter basis.

**Table 6:** Effects of dietary zinc supplementation on reproductive organs and follicle development of Chinese yellow-feathered broiler breeders aged 65 weeks.

Variable	Dietary Zinc Supplementation (mg/kg) ^1^						SEM ^2^	*p*-Value
	0	24	48	72	96	120		
Ovarian weight (g)	8.61	9.64	8.84	8.93	9.72	9.03	2.33	0.843
Oviduct weight (g)	54.43	54.20	51.10	56.12	58.57	56.22	1.57	0.782
Oviduct length (cm)	22.06	22.69	20.75	20.59	22.33	21.25	0.56	0.593
Total large follicle weight (g)	37.69	39.91	41.51	40.94	39.08	44.66	1.87	0.261
Large follicle number	4.25	4.17	4.90	4.83	4.42	4.82	0.16	0.243
Average large follicle weight (g)	8.91	9.87	8.30	8.55	8.84	9.36	0.38	0.636

The number of replicates was used as the experimental unit (*n* = 6). ^1^ The “0” treatment contained 22.81 mg zinc/kg, and the other zinc levels are additional to that level. ^2^ Standard error of the mean from ANOVA (*n* = 6).

**Table 7:** Effects of dietary zinc supplementation on liver and serum biochemical indices of yellow-feathered broiler breeders aged 65 weeks.

Variable	Dietary Zinc Supplementation (mg/kg) ^1^						SEM ^2^	*p*-Value
	0	24	48	72	96	120		
Serum								
CuZnSOD ^3^ (U/mL)	247.52 ^b,c^	219.99 ^c^	247.58 ^b,c^	295.20 ^a^	273.07 ^a,b^	238.61 ^c^	5.13	<0.001
MDA ^4^ (nmol/mL)	4.13 ^a^	3.58 ^a,b^	3.07 ^b^	3.33 ^b^	3.38 ^b^	3.15 ^b^	0.17	0.018
T-AOC ^5^ (U/mL)	4.59 ^b^	4.38 ^b^	6.56 ^a^	5.13 ^b^	5.09 ^b^	4.38 ^b^	0.18	0.004
AKP ^6^ (U/L)	1088.46	1147.59	1103.81	1395.81	146.75	1402.37	59.36	0.308
Zinc content (μmol /L)	27.68 ^b^	29.70^b^	30.39 ^b^	33.73 ^b^	42.08 ^a^	34.48 ^b^	7.94	0.031
GSH ^7^ content (mg/L)	10.39	10.36	10.12	10.46	9.25	9.49	0.58	0.154
GSH-Px ^8^ activity (U/L)	2294.84	2398.39	2344.18	2337.34	2363.46	2281.17	43.14	0.974
Liver								
CuZnSOD ^3^ (U/mg protein)	321.44 ^c^	362.21 ^b,c^	380.01 ^a,b^	380.18 ^a,b^	395.36 ^a,b^	415.94 ^a^	7.81	0.006
MDA ^4^ (nmol/mg protein)	0.45	0.44	0.47	0.46	0.46	0.46	0.019	0.998

In the same row, means not sharing a similar superscript (^a,b,c^) differ significantly (*p* < 0.05), the number of replicates was used as the experimental unit (*n* = 6). ^1^ The “0” treatment contained 22.81 mg zinc/kg, and the other zinc levels are additional to that level. ^2^ Standard error of the mean from ANOVA (*n* = 6). ^3^ CuZnSOD, copper-zinc superoxide dismutase. ^4^ MDA: malondialdehyde. ^5^ T-AOC, total antioxidant capacity. ^6^ AKP, alkaline phosphatase. ^7^ GSH, glutathione. ^8^ GSH-PX, Glutathione peroxidase.

**Table 8:** Effects of dietary zinc supplementation on the ovarian biochemical indices of yellow-feathered broiler breeders aged 65 weeks.

Item	Dietary Zinc Supplementation (mg/kg) ^1^						SEM ^2^	*p*-Value
	0	24	48	72	96	120		
CuZnSOD ^3^ (U/mg protein)	50.26 ^b^	53.08 ^b^	64.95 ^a,b^	53.87 ^b^	76.11 ^a^	80.20 ^a^	3.14	0.003
MDA ^4^ (nmol/mg protein)	0.39	0.34	0.33	0.36	0.33	0.32	0.036	0.637
T-AOC ^5^ (U/mg protein)	0.25	0.31	0.30	0.36	0.39	0.35	0.023	0.167
GSH ^6^ content (mg/g protein)	5.29	6.72	6.73	5.65	5.63	5.33	0.36	0.551
GSH-Px ^7^ activity (U/mg protein)	44.83 ^b^	43.59 ^b^	58.98 ^a,b^	59.81 ^a,b^	76.36 ^a^	77.86 ^a^	3.09	0.001

In the same row, means not sharing a similar superscript (^a,b^) differ significantly (*p* < 0.05), the number of replicates was used as the experimental unit (*n* = 6). ^1^ The “0” treatment contained 22.81 mg zinc/kg, and the other zinc levels are additional to that level. ^2^ Standard error of the mean from ANOVA (*n* = 6). ^3^ CuZnSOD, copper-zinc superoxide dismutase. ^4^ MDA: malondialdehyde. ^5^ T-AOC, total antioxidant capacity. ^6^ GSH, glutathione. ^7^ GSH-PX, Glutathione peroxidase.

## Abbreviations

The following abbreviations are used in this manuscript:

AKP	alkaline phosphatase
BW	Average body weight (g)
EA	Exponential asymptotic
ELR	Egg laying rate (%)
EM	Daily egg mass (g/bird)
FCR	Feed conversion ratio (g feed: g egg)
GSH	Glutathione
GSH-PX	glutathione peroxidase
MDA	malondialdehyde
QP	Quadratic polynomial
T-AOC	total antioxidant capacity
Wk	Week
